# ﻿Taxonomy and phylogeny of Auriculariales (Agaricomycetes, Basidiomycota) with descriptions of four new species from south-western China

**DOI:** 10.3897/mycokeys.108.128659

**Published:** 2024-08-30

**Authors:** Junhong Dong, Yonggao Zhu, Chengbin Qian, Changlin Zhao

**Affiliations:** 1 The Key Laboratory of Forest Resources Conservation and Utilization in the South-west Mountains of China Ministry of Education, Key Laboratory of National Forestry and Grassland Administration on Biodiversity Conservation in Southwest China, Yunnan Provincial Key Laboratory for Conservation and Utilization of In-forest Re-source, Southwest Forestry University, Kunming 650224, China Southwest Forestry University Kunming China; 2 College of Forestry, Southwest Forestry University, Kunming 650224, China Southwest Forestry University Kunming China

**Keywords:** Biodiversity, molecular systematics, taxonomy, wood-inhabiting fungi, Yunnan Province

## Abstract

The wood-inhabiting fungi play an integral role in wood degradation and the cycle of matter in the ecological system. They are considered as the “key player” in wood decomposition, because of their ability to produce lignocellulosic enzymes that break down woody lignin, cellulose and hemicellulose. In the present study, four new wood-inhabiting fungal species, *Adustochaetealbomarginata*, *Ad.punctata*, *Alloexidiopsisgrandinea* and *Al.xantha* collected from southern China, are proposed, based on a combination of morphological features and molecular evidence. *Adustochaetealbomarginata* is characterised by resupinate basidiomata with cream to buff, a smooth, cracked, hymenial surface, a monomitic hyphal system with clamped generative hyphae and subcylindrical to allantoid basidiospores (12–17.5 × 6.5–9 µm). *Adustochaetepunctata* is characterised by resupinate basidiomata with cream, a smooth, punctate hymenial surface, a monomitic hyphal system with clamped generative hyphae and subcylindrical to allantoid basidiospores (13.5–18 × 6–8.2 µm). *Alloexidiopsisgrandinea* is characterised by resupinate basidiomata with buff to slightly yellowish, a grandinioid hymenial surface, a monomitic hyphal system with clamped generative hyphae and allantoid basidiospores (10–12.3 × 5–5.8 µm). Additionally, *Alloexidiopsisxantha* is characterised by resupinate basidiomata with cream to slightly buff, a smooth hymenial surface, a monomitic hyphal system with clamped generative hyphae and subcylindrical to allantoid basidiospores measuring 20–24 × 5–6.2 µm. Sequences of the internal transcribed spacers (ITS) and the large subunit (nrLSU) of the nuclear ribosomal DNA (rDNA) markers of the studied samples were generated. Phylogenetic analyses were performed with the Maximum Likelihood, Maximum Parsimony and Bayesian Inference methods. The phylogram, based on the ITS+nLSU rDNA gene regions, revealed that four new species were assigned to the genera *Adustochaete* and *Alloexidiopsis* within the order Auriculariales, individually. The phylogenetic tree inferred from the ITS sequences highlighted that *Ad.albomarginata* was retrieved as a sister to *Ad.yunnanensis* and the species *Ad.punctata* was sister to *Ad.rava*. The topology, based on the ITS sequences, showed that *Al.grandinea* was retrieved as a sister to *Al.schistacea* and the taxon *Al.xantha* formed a monophyletic lineage. Furthermore, two identification keys to *Adustochaete* and *Alloexidiopsis* worldwide are provided.

## ﻿Introduction

In forest ecosystems, fungi play an essential ecological role to drive carbon cycling in forest soils, mediate mineral nutrition of plants and alleviate carbon limitations (Tedersoo et al. 2014). The fungal order Auriculariales is a group mainly composed of wood-inhabiting fungi in Agaricomycetes Doweld (Basidiomycota) (Hibbett et al. 2007). The type genus of this order is *Auricularia* Bull., in which several other gelatinous genera *Exidia* Fr., *Guepinia* Fr. and *Pseudohydnum* P. Karst., comprise important edible and medicinal fungi (Wu et al. 2019; Liu et al. 2022). Therefore, interest in species diversity in gelatinous genera has increased significantly in recent years (Chen et al. 2020; Shen and Fan 2020; Ye et al. 2020; Wang and Thorn 2021; Wu et al. 2021; Tohtirjap et al. 2023).

Contrary to the gelatinous genera, most species in the order Auriculariales are tough, include saprophytic species with resupinate, effused-reflexed, hydnoid, cerebriform, coralloid or pileate basidiomata (Wells and Bandoni 2001; Miettinen et al. 2012; Hibbett et al. 2014; Malysheva and Spirin 2017; Alvarenga et al. 2019; Spirin et al. 2019a, 2019b; Liu et al. 2022; Tohtirjap et al. 2023). Species with the stereoid basidiocarps are widely distributed in many orders of the Agaricomycetes, although they are certainly a minority in the order Auriculariales (Malysheva and Spirin 2017).

The genus *Adustochaete* Alvarenga & K.H. Larss. was erected by Alvarenga and Larsson and typed by the taxon *Ad.rava* Alvarenga & K.H. Larss. It is characterised by the resupinate basidiomata, spiny or tuberculate hymenophore, a monomitic hyphal structure with clamp connections on generative hyphae, present cystidia and hyphidia, ellipsoid-ovoid to obconical basidia, cylindrical to broadly cylindrical, straight or curved basidiospores (Alvarenga et al. 2019). The genus *Alloexidiopsis* L.W. Zhou & S.L. Liu is typified by *Al.schistacea* L.W. Zhou & S.L. Liu, which is c characterised by annual, resupinate basidiomata, smooth or with sterile spines hymenophore, a monomitic hyphal structure with clamp connections on generative hyphae, present cystidia and hyphidia, ellipsoid to ovoid, septate basidia, and cylindrical to broadly cylindrical, slightly curved (allantoid) basidiospores (Liu et al. 2022). Based on the MycoBank database (http://www.mycobank.org, accessed on 25 July 2024) and the Index Fungorum (http://www.indexfungorum.org, accessed on 25 July 2024), the genera *Adustochaete* and *Alloexidiopsis* have registered four and six species, respectively (Alvarenga et al. 2019; Guan et al. 2020; Hyde et al. 2020; Li et al. 2022a, 2022b; Li and Zhao 2022; Liu et al. 2022; Dong et al. 2024).

Classification of the kingdom of fungi has been updated continuously, based on the frequent inclusion of data from DNA sequences in many phylogenetic studies (Wijayawardene et al. 2020, 2022). Based on the early embrace of molecular systematics by mycologists, both the discovery and classification of fungi, amongst the more basal branches of the tree, are now coming to light from genomic analyses and environmental DNA surveys that have been conducted (James et al. 2020). Based on both the morphological and phylogenetic evidence, the generic concepts of *Eichleriella* Bres., *Hirneolina* (Pat.) Bres. and *Tremellochaete* Raitv. were revised, in which Malysheva and Spirin (2017) proposed that the genus *Heteroradulum* Lloyd ex Spirin and Malysheva was validated. The genus *Eichleriella* was accepted to be a monophyletic genus, while both genera *Exidiopsis* (Bref.) Möller and *Heterochaete* Pat. seemed to be synonymous, with priority given to the latter genus (Malysheva and Spirin 2017; Alvarenga et al. 2019; Alvarenga and Gibertoni 2021). However, certain species of *Exidiopsis*, even sequenced ones such as *E.calcea* (Pers.) K. Wells and *E.grisea* (Bres.) Bourdot & Maire, still have no appropriate placement at the generic level (Malysheva and Spirin 2017; Li et al. 2022a; Liu et al. 2022).

In recent years, the species diversity of the resupinate Auriculariales have been described or better defined using morphological and molecular analyses and the results showed the hidden diversity of this group and several corticioid genera, for example, *Adustochaete*, *Alloexidiopsis*, *Amphistereum* Spirin & Malysheva, *Crystallodon* Alvarenga, *Heteroradulum*, *Metulochaete* Alvarenga, *Proterochaete* Spirin & Malysheva and *Sclerotrema* Spirin & Malysheva, which have been established and described, based on the morphological and phylogenetic studies (Malysheva and Spirin 2017; Alvarenga et al. 2019; Spirin et al. 2019a, 2019b; Alvarenga and Gibertoni 2021; Liu et al. 2022).

During investigations on wood-inhabiting fungi in the Yunnan-Guizhou Plateau, China, many specimens were collected. To clarify the placement and relationships of these specimens, we carried out a phylogenetic and taxonomic study, based on the ITS+nLSU and ITS sequences. These specimens were assigned to the genera *Adustochaete* and *Alloexidiopsis* within the order Auriculariales. Therefore, four new species *Ad.albomarginata*, *Ad.punctata*, *Al.grandinea* and *Al.xantha* are proposed with description and illustrations, based on the morphological characteristics and phylogenetic analyses.

## ﻿Materials and methods

### ﻿Sample collection and herbarium specimen preparation

The fresh fruiting bodies were collected on the fallen angiosperm branches from Dali, Dehong, Diqing, Lincang and Zhaotong of Yunnan Province, China. The samples were photographed in situ and fresh macroscopic details were recorded. Photographs were recorded by a Nikon D7100 camera. All the photos were focus-stacked using Helicon Focus software. Macroscopic details were recorded and transported to a field station where the fruit body was dried on an electronic food dryer at 45 °C. Once dried, the specimens were sealed in an envelope and zip-lock plastic bags and labelled (Zhang et al. 2024). The dried specimens were deposited in the Herbarium of the Southwest Forestry University (SWFC), Kunming, Yunnan Province, China.

### ﻿Morphology

The macromorphological descriptions were based on field notes and photos captured in the field and lab. The colour terminology follows Petersen (1996). The micromorphological data were obtained from the dried specimens after observation under a light microscope with a magnification of 10 × 100 oil (Zhao et al. 2023). Sections mounted in 5% potassium hydroxide (KOH) and 2% phloxine B dye (C_20_H_2_Br_4_C_l4_Na_2_O_5_) and we also used other reagents, including Cotton Blue and Melzer’s reagent to observe micromorphology following Wu et al. (2022b). To show the variation in spore sizes, 5% of measurements were excluded from each end of the range and shown in parentheses. At least thirty basidiospores from each specimen were measured. Stalks were excluded from basidia measurements and the hilar appendage was excluded from basidiospores measurements. The following abbreviations are used: KOH = 5% potassium hydroxide water solution, CB– = acyanophilous, IKI– = both inamyloid and non-dextrinoid, L = mean spore length (arithmetic average for all spores), W = mean spore width (arithmetic average for all spores), Q = variation in the L/W ratios between the specimens studied, Q_m_ represented the average Q of basidiospores measured ± standard deviation and n = a/b (number of spores (a) measured from given number (b) of specimens).

### ﻿Molecular phylogeny

The CTAB rapid plant genome extraction kit-DN14 (Aidlab Biotechnologies Co., Ltd., Beijing, China) was used to obtain genomic DNA from the dried specimens according to the manufacturer’s instructions. The ITS region was amplified with ITS5 and ITS4 primers (White et al. 1990). The nLSU region was amplified with the LR0R and LR7 (Vilgalys and Hester 1990; Rehner and Samuels 1994). The PCR procedure for ITS was as follows: initial denaturation at 95 °C for 3 min, followed by 35 cycles at 94 °C for 40 s, 58 °C for 45 s and 72 °C for 1 min and a final extension of 72 °C for 10 min. The PCR procedure for nLSU was as follows: initial denaturation at 94 °C for 1 min, followed by 35 cycles at 94 °C for 30 s, 48 °C for 1 min and 72 °C for 1.5 min and a final extension of 72 °C for 10 min. The PCR products were purified and sequenced at Kunming Tsingke Biological Technology Limited Company (Yunnan Province, P.R. China). The newly-generated sequences were deposited in NCBI GenBank (Table 1).

**Table 1.** List of species, specimens, and GenBank accession number of sequences used in this study.

Species Name	Sample No.	GenBank Accession No.		Country	References
		ITS	nLSU		
***Adustochaetealbomarginata***	**CLZhao 22774** *	**PP852049**	**PP849033**	**China**	**Present study**
*Adustochaeteinterrupta*	LR 23435	MK391518	MK391527	Brazil	Alvarenga et al. (2019)
*Adustochaetenivea*	RLMA 531	MN165954	MN165989	USA	Liu et al. (2022)
***Adustochaetepunctata***	**CLZhao 29669**	**PP852050**	—	**China**	**Present study**
***Adustochaetepunctata***	**CLZhao 29671**	**PP852051**	**PP849034**	**China**	**Present study**
***Adustochaetepunctata***	**CLZhao 29675** *	**PP852052**	**PP849035**	**China**	**Present study**
***Adustochaetepunctata***	**CLZhao 29685**	**PP852053**	**PP849036**	**China**	**Present study**
***Adustochaetepunctata***	**CLZhao 29686**	**PP852054**	**PP849037**	**China**	**Present study**
***Adustochaetepunctata***	**CLZhao 29706**	**PP852055**	—	**China**	**Present study**
***Adustochaetepunctata***	**CLZhao 29710**	**PP852056**	**PP849038**	**China**	**Present study**
***Adustochaetepunctata***	**CLZhao 29711**	**PP852057**	**PP849039**	**China**	**Present study**
*Adustochaeterava*	RC 841	MK391516	—	Brazil	Alvarenga et al. (2019)
*Adustochaeterava*	KHL 15526	MK391517	MK391526	Brazil	Alvarenga et al. (2019)
*Adustochaeteyunnanensis*	CLZhao 8212	MZ911964	MZ950629	China	Li and Zhao (2022)
*Adustochaeteyunnanensis*	CLZhao 4671	MZ911965	—	China	Li and Zhao (2022)
*Adustochaeteyunnanensis*	CLZhao 4401	MZ911966	MZ950630	China	Li and Zhao (2022)
*Alloexidiopsisaustraliensis*	LWZ 20180514-18	OM801934	OM801919	China	Liu et al. (2022)
*Alloexidiopsisaustraliensis*	LWZ 20180513-22	OM801933	OM801918	China	Liu et al. (2022)
*Alloexidiopsiscalcea*	LWZ 20180904-14	OM801935	OM801920	China	Liu et al. (2022)
*Alloexidiopsiscalcea*	MW 331	AF291280	AF291326	Germany	Weiß and Oberwinkler (2001)
***Alloexidiopsisgrandinea***	**CLZhao 33798** *	**PP852058**	—	**China**	**Present study**
***Alloexidiopsisgrandinea***	**CLZhao 34279**	**PP852059**	—	**China**	**Present study**
*Alloexidiopsisnivea*	CLZhao 11204	MZ352947	MZ352938	China	Li et al. (2022a)
*Alloexidiopsisnivea*	CLZhao 11210	MZ352948	MZ352939	China	Li et al. (2022a)
*Alloexidiopsisschistacea*	LWZ 20200819-21a	OM801939	OM801932	China	Liu et al. (2022)
***Alloexidiopsisxantha***	**CLZhao 25093** *	**PP852060**	**PP849040**	**China**	**Present study**
*Alloexidiopsisyunnanensis*	CLZhao 8106	MT215569	MT215565	China	Guan et al. (2020)
*Alloexidiopsisyunnanensis*	CLZhao 4023	MT215568	MT215564	China	Guan et al. (2020)
*Amphistereumleveilleanum*	FP-106715	KX262119	KX262168	USA	Malysheva and Spirin (2017)
*Amphistereumschrenkii*	HHB 8476	KX262130	KX262178	USA	Malysheva and Spirin (2017)
*Aporpiumcaryae*	Miettinen 14774	JX044145	—	Finland	Miettinen et al. (2012)
*Aporpiumcaryae*	WD 2207	AB871751	AB871730	Japan	Sotome et al. (2014)
*Auriculariaauricula-judae*	JT 04	KT152099	KT152115	UK	Tohtirjap et al. (2023)
*Auriculariacornea*	Dai 13621	MZ618936	MZ669905	China	Tohtirjap et al. (2023)
*Auriculariapolytricha*	TUFC 12920	AB871752	AB871733	Japan	Sotome et al. (2014)
*Auriculariatibetica*	Dai 13336	MZ618943	MZ669915	China	Tohtirjap et al. (2023)
*Bourdotiagalzinii*	Otto MiettinenX3067	MG757511	MG757511	Spain	Malysheva et al. (2018)
*Crystallodonsubgelatinosum*	RC 1609-URM93444	MN475884	MN475888	Brazil	Alvarenga and Gibertoni (2021)
*Crystallodonsubgelatinosum*	TBG BF-18001-URM93445	MN475885	MN475889	Brazil	Alvarenga and Gibertoni (2021)
*Ductiferasucina*	KW3886	AY509551	AY509551	Canada	Liu et al. (2022)
*Eichleriellabactriana*	TAAM 55071	KX262121	KX262170	Russia	Malysheva and Spirin (2017)
*Eichleriellacrocata*	TAAM 101077	KX262100	KX262147	Russia	Malysheva and Spirin (2017)
*Eichleriellaleucophaea*	Barsukova LE 303261	KX262111	KX262161	Russia	Malysheva and Spirin (2017)
*Eichleriellatenuicula*	ValCB 1	MK391515	MK391525	Brazil	Alvarenga et al. (2019)
*Elmerinacladophora*	Miettinen 14314	MG757509	MG757509	Indonesia	Malysheva et al. (2018)
*Elmerinasclerodontia*	Miettinen 16431	MG757512	MG757512	Malaysia	Malysheva et al. (2018)
*Exidiaglandulosa*	YC Dai 21232	MT663362	MT664781	China	Wu et al. (2020)
*Exidiaglandulosa*	YC Dai 21233	MT663363	MT664782	China	Wu et al. (2020)
*Exidiapithya*	MW 313	AF291275	AF291321	Germany	Weiß and Oberwinkler (2001)
*Grammatuslabyrinthinus*	Yuan 1600	KM379139	KM379140	China	Alvarenga et al. (2019)
*Grammatussemis*	OM10618	KX262146	KX262194	China	Malysheva and Spirin (2017)
*Heteroradulumadnatum*	LR 23453	KX262116	KX262165	Mexico	Tohtirjap et al. (2023)
*Heteroradulumkmetii*	VS 6466	KX262104	KX262152	Russia	Malysheva and Spirin (2017)
*Hyalodonpiceicola*	Spirin 2689	MG735414	MG735422	Russia	Spirin et al. (2019a)
*Hyalodonpiceicola*	Spirin 11063	MG735415	MG735423	Russia	Spirin et al. (2019a)
*Mycostillavermiformis*	Spirin 11330	MG735417	MG735425	Russia	Spirin et al. (2019a)
*Mycostillavermiformis*	OF 188059	MG735418	—	Russia	Spirin et al. (2019a)
*Myxariumcinnamomescens*	OF160494	KY801882	KY801909	Russia	Spirin et al. (2018)
*Myxariumgrilletii*	VS9016	MK098896	MK098944	Russia	Spirin et al. (2019b)
*Myxariumhyalinum*	TL2012 443455	KY801880	KY801907	Russia	Spirin et al. (2018)
*Myxariumlegonii*	VS 8986	MK098899	MK098947	Russia	Spirin et al. (2019b)
*Protodaedaleafoliacea*	Miettinen 13 054	MG757507	MG757507	Finland	Malysheva et al. (2018)
*Protodaedaleahispida*	Spirin 5139	MG757510	MG757510	Finland	Malysheva et al. (2018)
*Protodontiaafricana*	AS 171126 1104	MK098978	MK098973	Russia	Spirin et al. (2019b)
*Protohydnumcartilagineum*	SP 467240	MG735419	MG735426	Russia	Malysheva et al. (2018)
*Protomeruliusdubius*	VS 3019	MK484041	MK480553	Russia	Spirin et al. (2019a)
*Protomeruliusminor*	KHL 15937	MK484060	MK480569	Russia	Spirin et al. (2019a)
*Protomeruliussubstuppeus*	O 19171	JX134482	JQ764649	China	Spirin et al. (2019a)
*Pseudohydnumgelatinosum*	F14063	AF384861	AF384861	Canada	Weiß and Oberwinkler (2001)
*Pseudohydnumgelatinosum*	AFTOL ID1875	DQ520094	DQ520094	Germany	Lutzoni et al. (2004)
*Stypellopsisfarlowii*	Larsson 12337	MG857095	MG857099	Russia	Spirin et al. (2018)
*Stypellopsishyperborea*	J Norden 9751	MG857097	MG857101	Russia	Spirin et al. (2018)
*Tremellochaeteatlantica*	URM90199	MG594381	MG594383	Brazil	Alvarenga et al. (2019)
*Tremellochaetejaponica*	TAA 42689	AF291274	AF291320	Russia	Weiß and Oberwinkler (2001)
*Tremiscushelvelloides*	AFTOL ID1680	DQ520100	DQ520100	Germany	Lutzoni et al. (2004)
*Sistotremabrinkmannii*	isolate 236	JX535169	JX535170	Netherlands	Alvarenga and Gibertoni (2021)

New species is shown in bold; * is shown type material, holotype.

**Figure 1.**
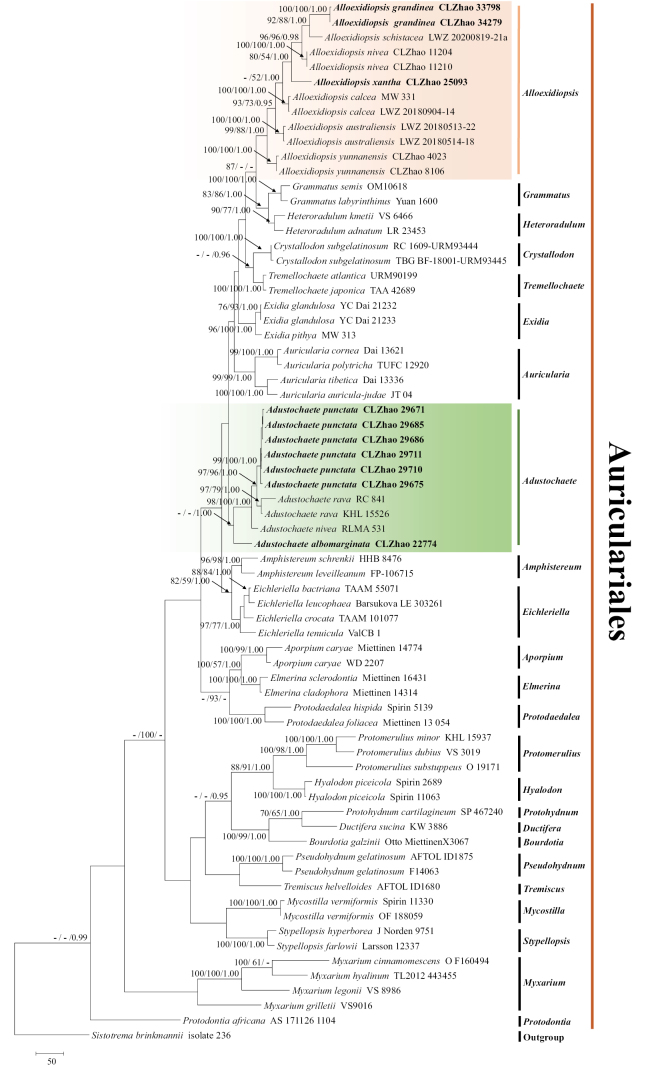
Maximum parsimony strict consensus tree illustrating the phylogeny of *Adustochaete* and *Alloexidiopsis* and related genera in the order Auriculariales, based on ITS+nLSU sequences. Branches are labelled with Maximum Likelihood bootstrap value ≥ 70%, parsimony bootstrap value ≥ 50% and Bayesian posterior probabilities ≥ 0.95.

**Figure 2.**
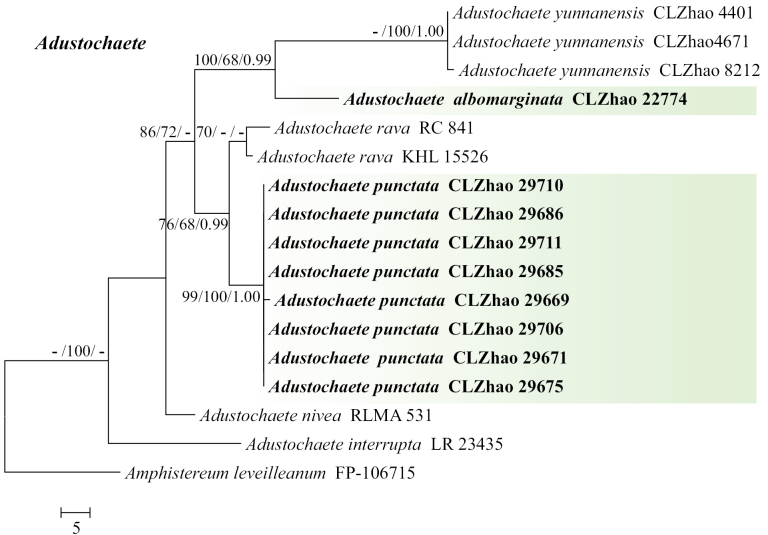
Maximum parsimony strict consensus tree illustrating the phylogeny of the two new species and related genera in the genus *Adustochaete*, based on ITS sequences. Branches are labelled with Maximum Likelihood bootstrap value ≥ 70%, parsimony bootstrap value ≥ 50% and Bayesian posterior probabilities ≥ 0.95.

**Figure 3.**
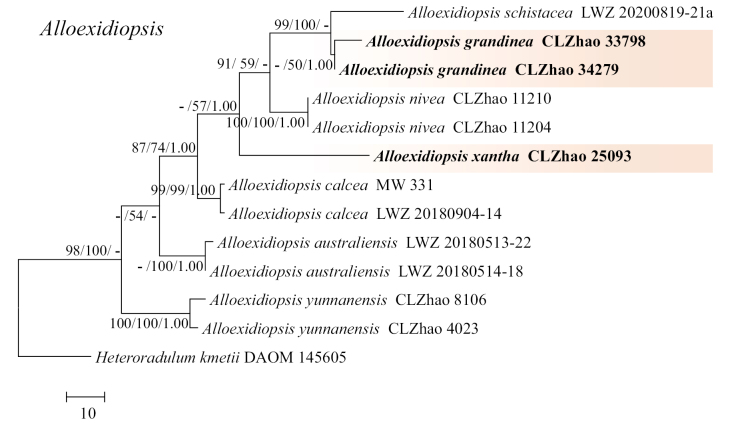
Maximum parsimony strict consensus tree illustrating the phylogeny of the two new species and related genera in the genus *Alloexidiopsis*, based on ITS sequences. Branches are labelled with Maximum Likelihood bootstrap value ≥ 70%, parsimony bootstrap value ≥ 50% and Bayesian posterior probabilities ≥ 0.95.

The sequences were aligned in MAFFT v. 7 (Katoh et al. 2019) using the G-INS-i strategy. The alignment was adjusted manually using AliView v. 1.27 (Larsson 2014). The dataset was aligned first and then the sequences of ITS+nLSU were combined with Mesquite v. 3.51. The combined ITS+nLSU sequences and ITS datasets were used to infer the position of the new species and related species. The sequence of *Sistotremabrinkmannii* (Bres.) J. Erikss. obtained from GenBank was used as an outgroup to root trees in the ITS+nLSU analysis (Fig. 1) in the order Auriculariales (Tohtirjap et al. 2023). The sequence of *Amphistereumleveilleanum* (Berk. & M.A. Curtis) Spirin & Malysheva obtained from GenBank was used as an outgroup to root trees in the ITS analysis in the genus *Adustochaete* (Fig. 2). The sequence of *Heteroradulumkmetii* (Bres.) Spirin & Malysheva obtained from GenBank was used as an outgroup to root trees in the ITS analysis in the genus *Alloexidiopsis* (Fig. 3).

Maximum Parsimony (MP), Maximum Likelihood (ML) and Bayesian Inference (BI) analyses were applied to the combined three datasets following a previous study (Zhao and Wu 2017) and the tree construction procedure was performed in PAUP* v. 4.0b10 (Swofford 2002). All of the characters were equally weighted and gaps were treated as missing data. Using the heuristic search option with TBR branch swapping and 1000 random sequence additions, trees were inferred. Maxtrees were set to 5000, branches of zero length were collapsed and all parsimonious trees were saved. Clade robustness was assessed using bootstrap (BT) analysis with 1000 replicates (Felsenstein 1985). Descriptive tree statistics, tree length (TL), the consistency index (CI), the retention index (RI), the rescaled consistency index (RC) and the homoplasy index (HI) were calculated for each maximum parsimonious tree generated. The multiple sequence alignment was also analysed using Maximum Likelihood (ML) in RAxML-HPC2 on XSEDE v. 8.2.8 with default parameters (Miller et al. 2012). Branch support (BS) for ML analysis was determined by 1000 bootstrap replicates.

jModelTest v. 2 (Darriba et al. 2012) was used to determine the best-fit evolution model for each dataset for the purposes of Bayesian Inference (BI), which was performed using MrBayes 3.2.7a with a GTR+I+G model of DNA substitution and a gamma distribution rate variation across sites (Ronquist et al. 2012). The first one-quarter of all the generations were discarded as burn-in. The majority-rule consensus tree of all the remaining trees was calculated. Branches were considered significantly supported if they received a Maximum Likelihood bootstrap value (BS) of > 70%, a Maximum Parsimony bootstrap value (BT) of > 70% or Bayesian Posterior Probabilities (BPP) of > 0.95.

## ﻿Results

### ﻿Sequence similarity search

The results of BLAST queries in NCBI, based on ITS and nLSU separately, showed the sequences producing significant alignment descriptions:

*Adustochaetealbomarginata*: in ITS BLAST results, *Ad.rava*, *Exidiasaccharina* Fr., *Ea.qinghaiensis* S.R. Wang & Thorn, *Ad.nivea* Alvarenga and *Exidiopsismucedinea* (Pat.) K. Wells were found as the top ten taxa (maximum record descriptions: Max score 830; Total score 830; Query cover 96%; E value 0.0; Ident 92.93%). In nLSU BLAST results, *Alloexidiopsisyunnanensis* (C.L. Zhao) L.W. Zhou & S.L. Liu, *Auriculariaasiatica* Bandara & K.D. Hyde, *Au.brasiliana* Y.C. Dai & F. Wu and *Steccherinumnandinae* (F. Wu, P. Du & X.M. Tian) Z.B. Liu, Y.C. Dai & Jing Si were found as the top ten taxa (maximum record descriptions: Max score 2398; Total score 2398; Query cover 98%; E value 0.0; Ident 98.60%).

*Adustochaetepunctata*: in ITS BLAST results, *Ad.rava*, *Ad.nivea*, *Exidiopsismucedinea* and *Exidiacandida* Lloyd were found as the top ten taxa (maximum record descriptions: Max score 959; Total score 959; Query cover 96%; E value 0.0; Ident 96.74%). In nLSU BLAST results, *Ad.rava*, *Ad.yunnanensis* Y.F. Li & C.L. Zhao., *Auriculariathailandica* Bandara & K.D. Hyde, *Au.scissa* Looney, Birkebak & Matheny, *Au.nigricans* (Sw.) Birkebak, Looney & Sánchez-García and *Alloexidiopsisyunnanensis* were found as the top ten taxa (maximum record descriptions: Max score 2464; Total score 2464; Query cover 98%; E value 0.0; Ident 99.34%).

*Alloexidiopsisgrandinea*: in ITS BLAST results, *Ad.nivea* and *Al.schistacea* were found as the top ten taxa (maximum record descriptions: Max score 861; Total score 861; Query cover 91%; E value 0.0; Ident 94.94%).

*Alloexidiopsisxantha*: in ITS BLAST results, *Al.sinensis* J.H. Dong & C.L. Zhao was found as the top ten taxa (maximum record descriptions: Max score 832; Total score 832; Query cover 98%; E value 0.0; Ident 92.42%). In nLSU BLAST results, *Al.sinensis* and *Al.yunnanensis* were found as the top ten taxa (maximum record descriptions: Max score 2457; Total score 2457; Query cover 99%; E value 0.0; Ident 99.05%).

The aligned dataset comprised 70 specimens representing 53 species. Four Markov chains were run for two runs from random starting trees, each for two million generations for the combine ITS+nLSU (Fig. 1) dataset with trees and parameters sampled every 1000 generations. The dataset had an aligned length of 2333 characters, of which 1301 characters are constant, 368 are variable and parsimony uninformative and 664 are parsimony informative. Maximum parsimony analysis yielded 120 equally parsimonious trees (TL = 4342, CI = 0.4000, HI = 0.6000, RI = 0.5288 and RC = 0.2115). The best model for the ITS+nLSU dataset, estimated and applied in the Bayesian analysis, was SYM+I+G. Both Bayesian analysis and ML analysis resulted in a similar topology to MP analysis with an average standard deviation of split frequencies = 0.008542 (BI) and the effective sample size (ESS) for Bayesian analysis across the two runs is double of the average ESS (avg. ESS) = 395.5.

The aligned dataset comprised 17 specimens representing seven species. Four Markov chains were run for two runs from random starting trees, each for 0.5 million generations for the ITS (Fig. 2) dataset with trees and parameters sampled every 1000 generations. The dataset had an aligned length of 522 characters, of which 413 characters are constant, 47 are variable and parsimony uninformative and 62 are parsimony informative. Maximum parsimony analysis yielded four equally parsimonious trees (TL = 161, CI = 0.8075, HI = 0.1925, RI = 0.8306 and RC = 0.6707). The best model for the ITS dataset, estimated and applied in the Bayesian analysis, was SYM+G. Both Bayesian analysis and ML analysis resulted in a similar topology to MP analysis with an average standard deviation of split frequencies = 0.006786 (BI) and the effective sample size (ESS) for Bayesian analysis across the two runs is double the average ESS (avg. ESS) = 617.

The aligned dataset comprised 13 specimens representing eight species. Four Markov chains were run for two runs from random starting trees, each for 0.3 million generations for the ITS (Fig. 3) dataset with trees and parameters sampled every 1000 generations. The dataset had an aligned length of 562 characters, of which 417 characters are constant, 64 are variable and parsimony uninformative and 81 are parsimony informative. Maximum parsimony analysis yielded two equally parsimonious trees (TL = 218, CI = 0.784, HI = 0.2156, RI = 0.7814 and RC = 0.6129). The best model for the ITS dataset, estimated and applied in the Bayesian analysis, was SYM+G. Both Bayesian analysis and ML analysis resulted in a similar topology to MP analysis with an average standard deviation of split frequencies = 0.007707 (BI) and the effective sample size (ESS) for Bayesian analysis across the two runs is double of the average ESS (avg. ESS) = 639.5.

The phylogram, based on the combined ITS+nLSU sequences (Fig. 1) analysis, showed that four new species *Ad.albomarginata*, *Ad.punctata*, *Al.grandinea* and *Al.xantha* were assigned to the genera *Adustochaete* and *Alloexidiopsis* within the order Auriculariales, individually. The phylogenetic tree, based on ITS sequences (Fig. 2), revealed that *Ad.albomarginata* was retrieved as a sister to *Ad.yunnanensis*. The taxon *Ad.punctata* was sister to *Ad.rava*. The topology, based on the ITS sequences (Fig. 3), revealed that *Al.grandinea* was retrieved as a sister to *Al.schistacea* and the species *Al.xantha* formed a monophyletic lineage.

### ﻿Taxonomy

#### ﻿ Adustochaete albomarginata

Taxon classificationFungiAuricularialesExidiaceae

J.H Dong & C.L. Zhao sp. nov.

FE1AB077-33E7-57BC-81AB-45982E09F628

854168

Figs 4 , 5 , 6

##### Diagnosis.

Differs from other *Adustochaete* species by its soft membranaceous basidiomata with cream to buff, smooth, cracked hymenial surface, a monomitic hyphal system with clamped generative hyphae and subcylindrical to allantoid basidiospores measuring 12–17.5 × 6.5–9 µm.

##### Holotype.

China • Yunnan Province, Dali, Weishan County, Leqiu Town, Zhongyao Village, 25°01′N, 100°19′E, altitude 1910 m, on the fallen branch of angiosperm, leg. C.L. Zhao, 19 July 2022, CLZhao 22774 (SWFC).

**Figure 4.**
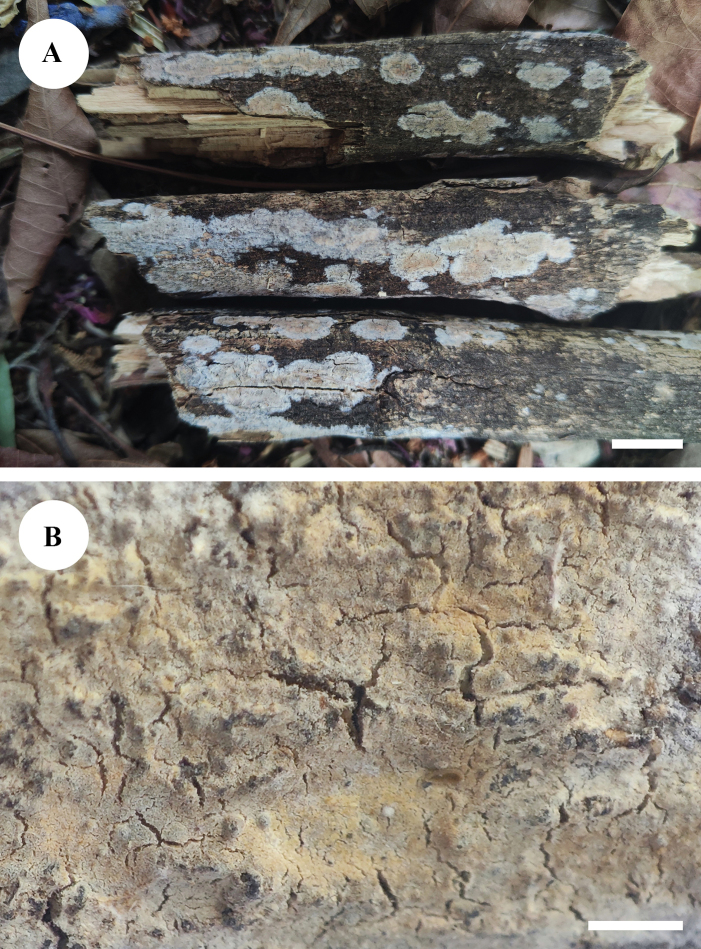
Basidiomata of *Adustochaetealbomarginata* in general and detailed views (CLZhao 22774, holotype). Scale bars: 1 cm (**A**); 1 mm (**B**).

##### Etymology.

*albomarginata* (Latin or Greek origin): referring to the white margin of the basidiomata.

##### Basidiomata.

Annual, resupinate, closely adnate, soft membranaceous, very hard to separate from substrate, without odour or taste when fresh, becoming coriaceous upon drying, up to 5 cm long, 1.5 cm wide, 50–100 µm thick. Hymenial surface smooth, white to cream when fresh, turning to cream to buff upon drying, cracked. Sterile margin white, thinning out, up to 0.5 mm wide.

**Figure 5.**
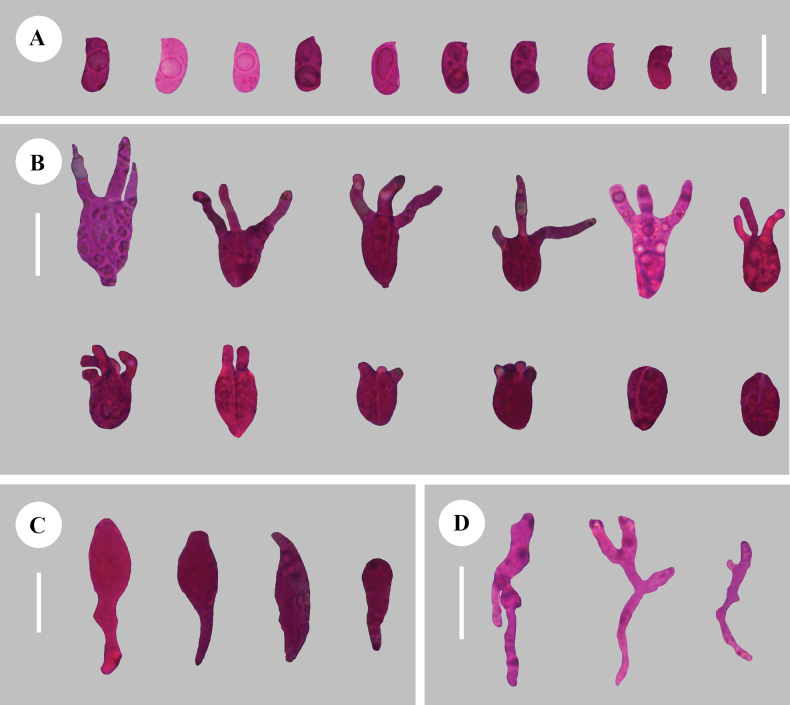
Sections of hymenium of *Adustochaetealbomarginata* (holotype, CLZhao 22774) **A** basidiospores **B** basidia **C** cystidia **D** hyphidia. Scale bars: 20 µm (**A–D**); 10 × 100 Oil.

##### Hyphal system.

Monomitic, generative hyphae with clamp connections, colourless, thin-walled, unbranched, interwoven, 2.5–3.5 µm in diameter; IKI–, CB–, tissues unchanged in KOH. ***Hymenium.*** Cystidia numerous, thin-walled, subclavate to fusiform with an acute or obtuse apex, occasionally sinuous in the basal, 23.5–48.5 × 10–13.5 µm, with a clamp connection at base; cystidioles absent. Hyphidia arising from generative hyphae, nodulose, branched, colourless, thin-walled, 2.5–5 µm in diameter. Basidia ellipsoid to ovoid, longitudinally septate, two to four-celled, 17–24.5 × 11–16.5 µm; basidioles dominant, similar to basidia in shape, but slightly smaller. ***Basidiospores.*** Subcylindrical to allantoid, slightly curved, colourless, smooth, thin-walled, with 1–2 oil drops, IKI–, CB–, (11.5–)12–17.5(–18) × 6.5–9(–9.5) µm, L = 14.66 µm, W = 7.80 µm, Q = 1.72–1.99, Q_m_ = 1.88 ± 0.08 (n = 30/1).

**Figure 6.**
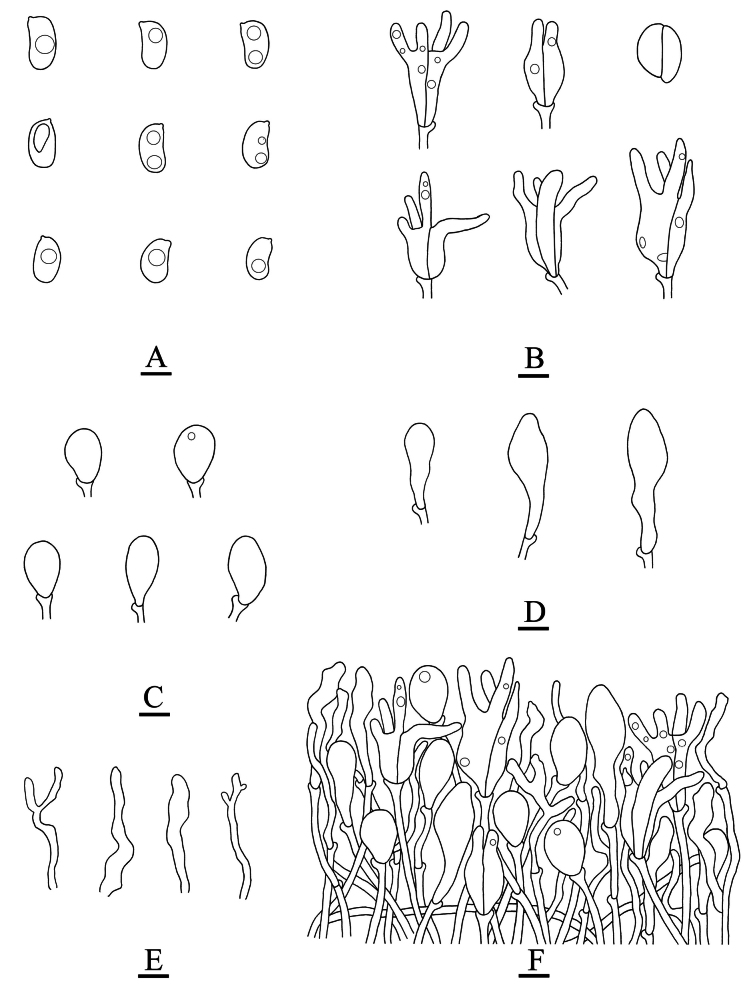
Microscopic structures of *Adustochaetealbomarginata* (holotype, CLZhao 22774) **A** basidiospores **B** basidia **C** basidioles **D** cystidia **E** hyphidia **F** part of the vertical section of hymenium. Scale bars: 10 µm (**A–F**).

#### ﻿ Adustochaete punctata

Taxon classificationFungiAuricularialesExidiaceae

J.H Dong & C.L. Zhao sp. nov.

7CFE9A66-C65B-593E-876E-BFC40F3BEA23

854170

Figs 7 , 8 , 9

##### Diagnosis.

Differs from other *Adustochaete* species by its membranaceous basidiomata with cream, smooth, punctate hymenial surface, a monomitic hyphal system with clamped generative hyphae and subcylindrical to allantoid basidiospores measuring 13.5–18 × 6–8.2 µm.

##### Holotype.

China • Yunnan Province, Dehong, Yingjiang County, Tongbiguan Provincial Nature Reserve, 23°48′N, 97°38′E, altitude 1500 m, on the fallen branch of angiosperm, leg. C.L. Zhao, 17 July 2023, CLZhao 29675 (SWFC).

##### Etymology.

*punctata* (Latin or Greek origin): referring to the punctate hymenial surface of the specimen.

##### Basidiomata.

Annual, resupinate, closely adnate, membranaceous, very hard to separate from substrate, without odour or taste when fresh, becoming coriaceous upon drying, up to 10 cm long, 1.5 cm wide, 100–250 µm thick. Hymenial surface smooth, punctate, white to cream when fresh, turning to cream upon drying. Sterile margin cream, thinning out, up to 1 mm wide.

**Figure 7.**
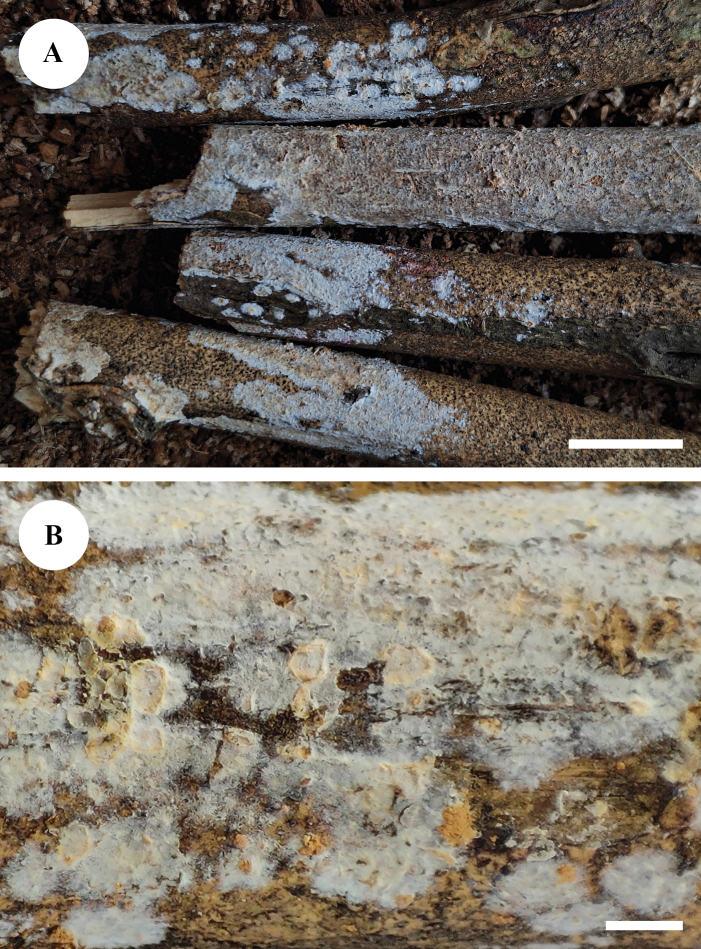
Basidiomata of *Adustochaetepunctata* in general and detailed views (CLZhao 29675, holotype). Scale bars: 1 cm (**A**); 1 mm (**B**).

##### Hyphal system.

Monomitic, generative hyphae with clamp connections, colourless, thin-walled, unbranched, interwoven, 1.5–3.5 µm in diameter; IKI–, CB–, tissues unchanged in KOH. ***Hymenium.*** Cystidia numerous, thin-walled, subcylindrical to clavate with an obtuse apex, occasionally sinuous in the basal, 15.5–23.5 × 5.5–7.5 µm, with a clamp connection at base; cystidioles absent. Hyphidia arising from generative hyphae, nodulose, branched, colourless, thin-walled, 1.5–5 μm in diameter. Basidia ellipsoid to ovoid, longitudinally septate, two to four-celled, 17–25 × 16.5–21 µm; basidioles dominant, similar to basidia in shape, but slightly smaller. ***Basidiospores.*** Subcylindrical to allantoid, slightly curved, colourless, smooth, thin-walled, with several oil drops, IKI–, CB–, (13–)13.5–18(–18.5) × (5.5–)6–8.2(–8.5) µm, L = 15.78 µm, W = 6.79 µm, Q = 2.15–2.40 Q_m_ = 2.32 ± 0.08 (n = 90/3).

**Figure 8.**
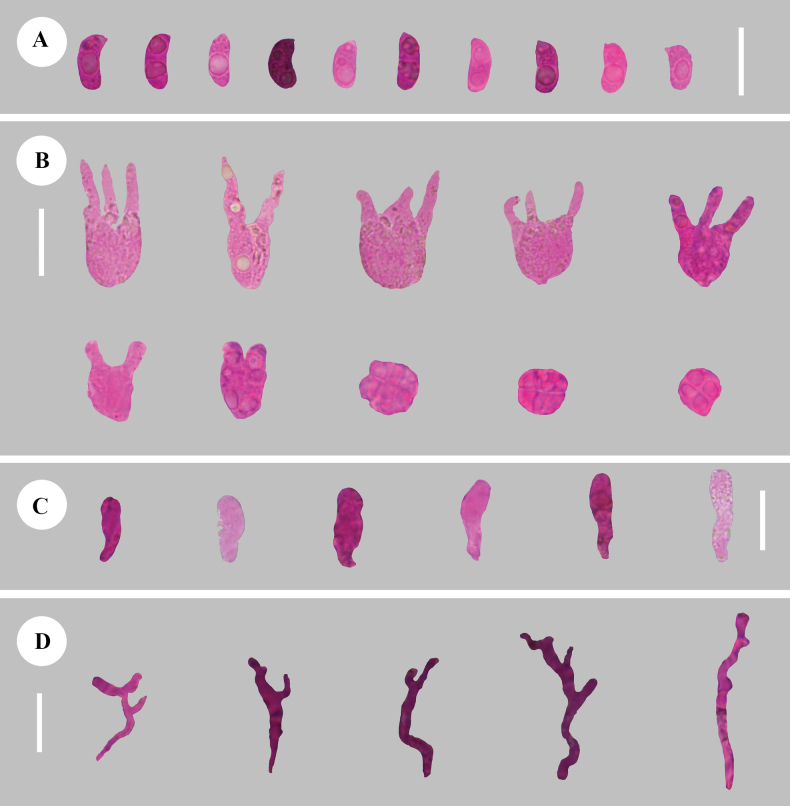
Sections of hymenium of *Adustochaetepunctata* (holotype, CLZhao 29675) **A** basidiospores **B** basidia **C** cystidia **D** hyphidia. Scale bars: 20 µm (**A–D**); 10 × 100 Oil.

##### Additional specimens examined.

China • Yunnan Province, Dehong, Yingjiang County, Tongbiguan Provincial Nature Reserve, 23°48′N, 97°38′E, altitude 1500 m, on the fallen branch of angiosperm, leg. C.L. Zhao, 17 July 2023, CLZhao 29669; CLZhao 29671; CLZhao 29685; CLZhao 29686; CLZhao 29706; CLZhao 29710; CLZhao 29711 (SWFC).

**Figure 9.**
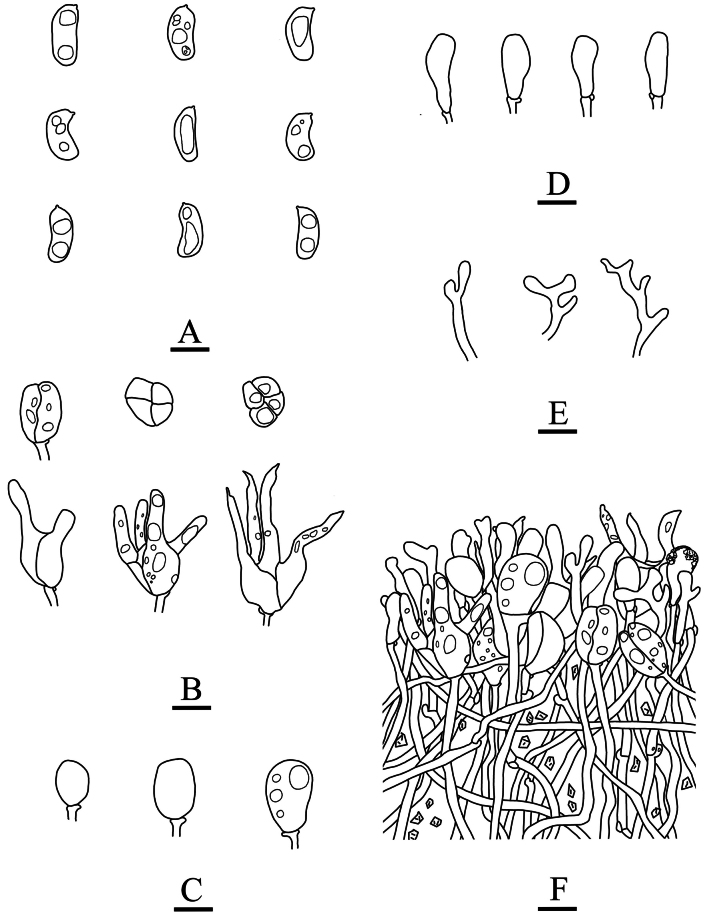
Microscopic structures of *Adustochaetepunctata* (holotype, CLZhao 29675) **A** basidiospores **B** basidia **C** basidioles **D** cystidia **E** hyphidia **F** part of the vertical section of hymenium. Scale bars: 10 µm (**A–F**).

#### ﻿ Alloexidiopsis grandinea

Taxon classificationFungiAuricularialesAuriculariaceae

J.H Dong & C.L. Zhao sp. nov.

E5A16478-6DE6-54DD-92AC-32E3CFE78F86

854171

Figs 10 , 11 , 12

##### Diagnosis.

Differs from other *Alloexidiopsis* species by its membranaceous basidiomata with buff to slightly yellowish, grandinioid hymenial surface, a monomitic hyphal system with clamped generative hyphae and cylindrical to allantoid basidiospores measuring 10–12.3 × 5–5.8 µm.

##### Holotype.

China • Yunnan Province, Zhaotong, Wumengshan National Nature Reserve, 28°03′N, 104°20′E, altitude 1500 m, on the fallen branch of angiosperm, leg. C.L. Zhao, 21 September 2023, CLZhao 33798 (SWFC).

##### Etymology.

*grandinea* (Latin or Greek origin): referring to the grandinioid hymenial surface.

##### Basidiomata.

Annual, resupinate, closely adnate, membranaceous, very hard to separate from substrate, without odour or taste when fresh, becoming coriaceous upon drying, up to 20 cm long, 3 cm wide, 50–100 µm thick. Hymenial surface grandinioid, white to buff when fresh, turning to buff to slightly yellowish upon drying. Sterile margin cream to buff, thinning out, up to 1 mm wide.

**Figure 10.**
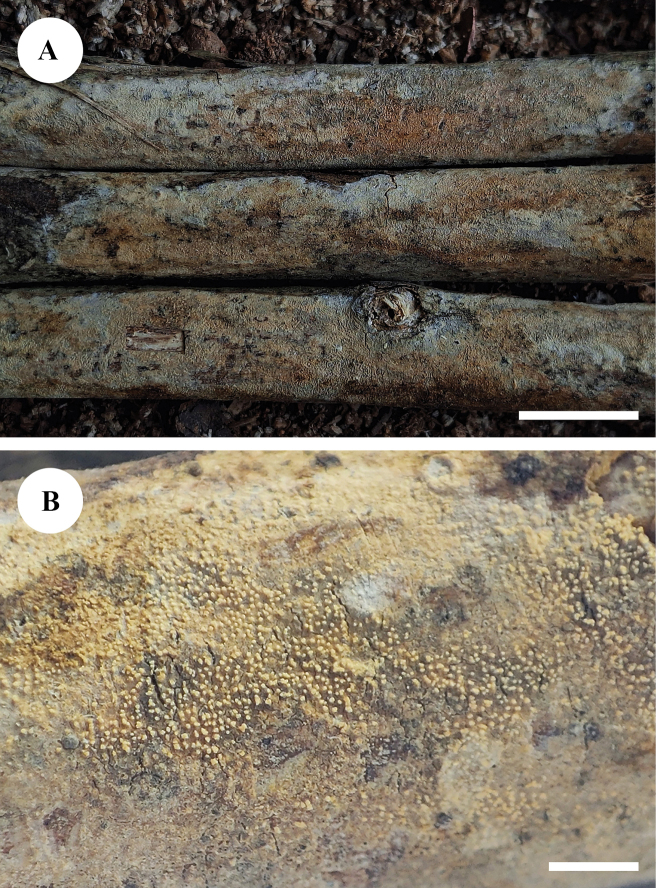
Basidiomata of *Alloexidiopsisgrandinea* in general and detailed views (CLZhao 33798, holotype). Scale bars: 1 cm (**A**); 1 mm (**B**).

##### Hyphal system.

Monomitic, generative hyphae with clamp connections, colourless, thin-walled, rarely branched, interwoven, 2–4 µm in diameter; IKI–, CB–, tissues unchanged in KOH. ***Hymenium.*** Cystidia numerous, thin-walled, fusiform with an acute apex, occasionally sinuous in the basal, 20–42.5 × 5.5–9.5 µm, with a clamp connection at base; cystidioles absent. Hyphidia arising from generative hyphae, nodulose, frequently branched, colourless, thin-walled, 2–5 µm in diameter. Basidia ellipsoid to ovoid, longitudinally septate, two to four-celled, 12.5–14.5 × 9–11.5 µm; basidioles dominant, similar to basidia in shape, but slightly smaller. ***Basidiospores.*** Cylindrical to allantoid, slightly curved, colourless, smooth, thin-walled, with 1–2 oil drops, IKI–, CB–, (9.5–)10–12.3(–12.5) × (4.8–)5–5.8(–6) µm, L = 11.08 µm, W = 5.38 µm, Q = 1.95–2.20, Q_m_ = 2.06 ± 0.04 (n = 60/2).

**Figure 11.**
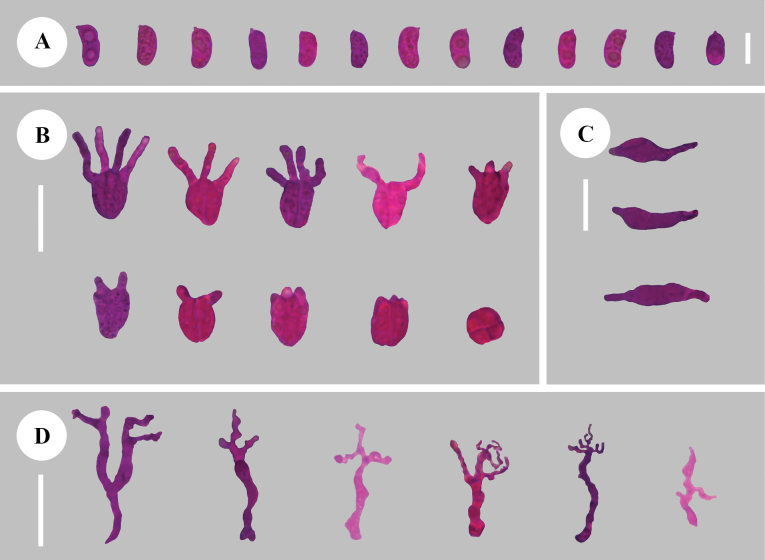
Sections of hymenium of *Alloexidiopsisgrandinea* (holotype, CLZhao 33798) **A** basidiospores **B** basidia **C** cystidia **D** hyphidia. Scale bars: 10 µm (**A**); 20 µm (**B–D**); 10 × 100 Oil.

##### Additional specimen examined.

China • Yunnan Province, Diqing, Weixi County, Weiden Town, Fuchuan Village, 27°06′N, 99°10′E, altitude 2900 m, on the fallen branch of angiosperm, leg. C.L. Zhao, 12 October 2023, CLZhao 34279 (SWFC).

**Figure 12.**
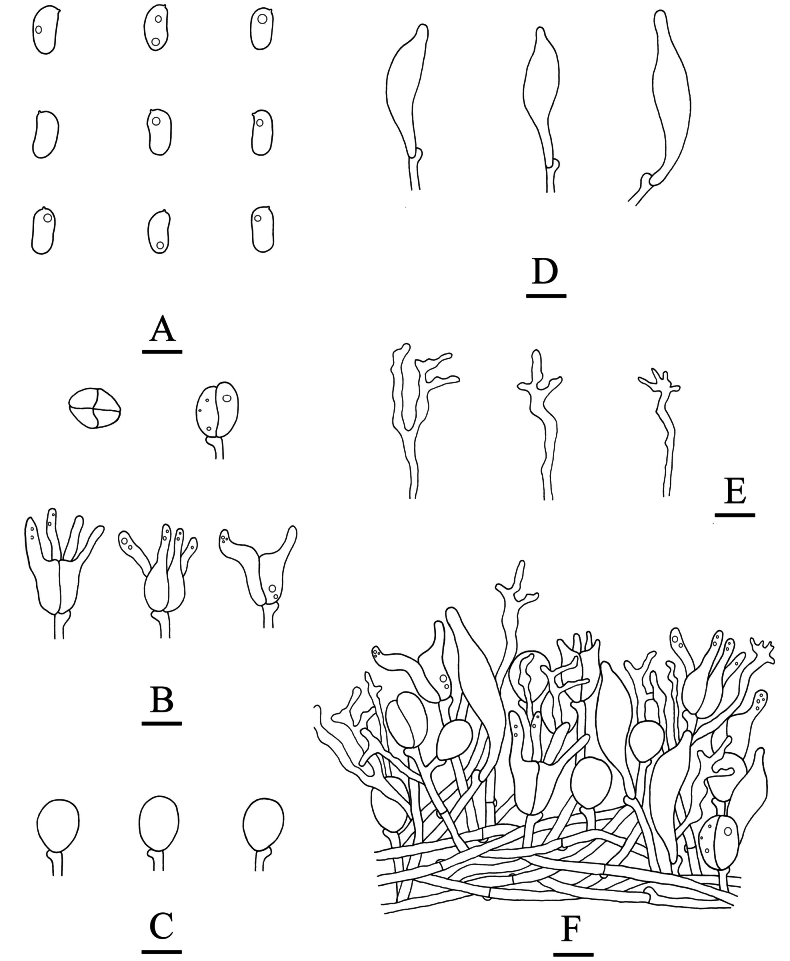
Microscopic structures of *Alloexidiopsisgrandinea* (holotype, CLZhao 33798) **A** basidiospores **B** basidia **C** basidioles **D** cystidia **E** hyphidia **F** part of the vertical section of hymenium. Scale bars: 10 µm (**A–F**).

#### ﻿ Alloexidiopsis xantha

Taxon classificationFungiAuricularialesAuriculariaceae

J.H. Dong & C.L. Zhao sp. nov.

41994D3C-BA51-5BCA-9B54-998A6CDECB73

854172

Figs 13 , 14 , 15

##### Diagnosis.

Differs from other *Alloexidiopsis* species by its coriaceous basidiomata with cream to buff to yellow, smooth, slightly cracked hymenial surface, a monomitic hyphal system with clamped generative hyphae and allantoid to sickle-shaped basidiospores measuring 20–24 × 5–6.2 µm.

##### Holotype.

China • Yunnan Province, Lincang, Yun County, Dumu Village, 24°32′N, 100°23′E, altitude 2100 m, on the fallen branch of angiosperm, leg. C.L. Zhao, 20 October 2022, CLZhao 25093 (SWFC).

##### Etymology.

*xantha* (Latin or Greek origin): referring to the buff to yellow hymenial surface of the type specimen.

##### Basidiomata.

Annual, resupinate, closely adnate, coriaceous, very hard to separate from substrate, without odour or taste when fresh, becoming leathery upon drying, up to 10 cm long, 2 cm wide, 200–300 µm thick. Hymenial surface smooth, slightly cracked, cream when fresh, turning to cream to buff to yellow upon drying. Sterile margin cream, thinning out, up to 1 mm wide.

**Figure 13.**
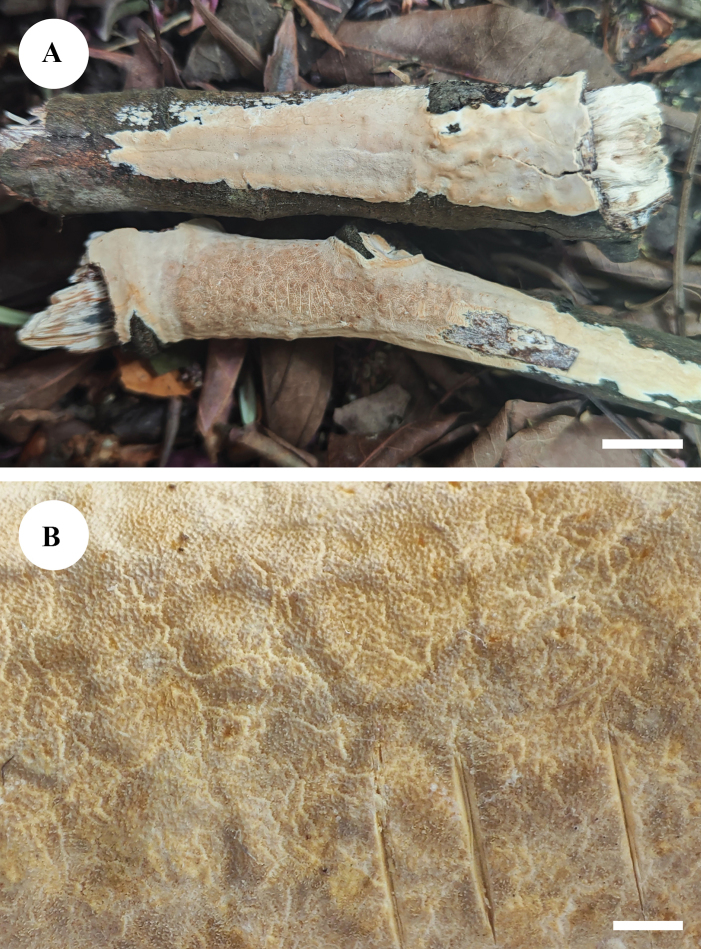
Basidiomata of *Alloexidiopsisxantha* in general and detailed views (CLZhao 25093, holotype). Scale bars: 1 cm (**A**); 1 mm (**B**).

##### Hyphal system.

Monomitic, generative hyphae with clamp connections, colourless, thin- to thick walled, branched, interwoven, 2.5–3.5 µm in diameter; IKI–, CB–, tissues unchanged in KOH. ***Hymenium.*** Cystidia numerous, thin-walled, subcylindrical to subconiform with an obtuse apex, 12.5–17.5 × 3.5–6 µm, with a clamp connection at base; cystidioles absent. Hyphidia arising from generative hyphae, nodulose, frequently branched, colourless, thin-walled, 2.5–4 µm in diameter. Basidia ellipsoid to ovoid, obconical, longitudinally septate, two to four-celled, 18–20.5 × 12–15.5 µm; basidioles dominant, similar to basidia in shape, but slightly smaller. ***Basidiospores.*** Allantoid, curved, sickle-shaped, colourless, smooth, thin-walled, IKI–, CB–, (18.5–)20–24(–24.5) × 5–6.2(–6.5) µm, L = 21.66 µm, W = 5.63 µm, Q = 3.60–4.05, Q_m_ = 3.85 ± 0.10 (n = 30/1).

**Figure 14.**
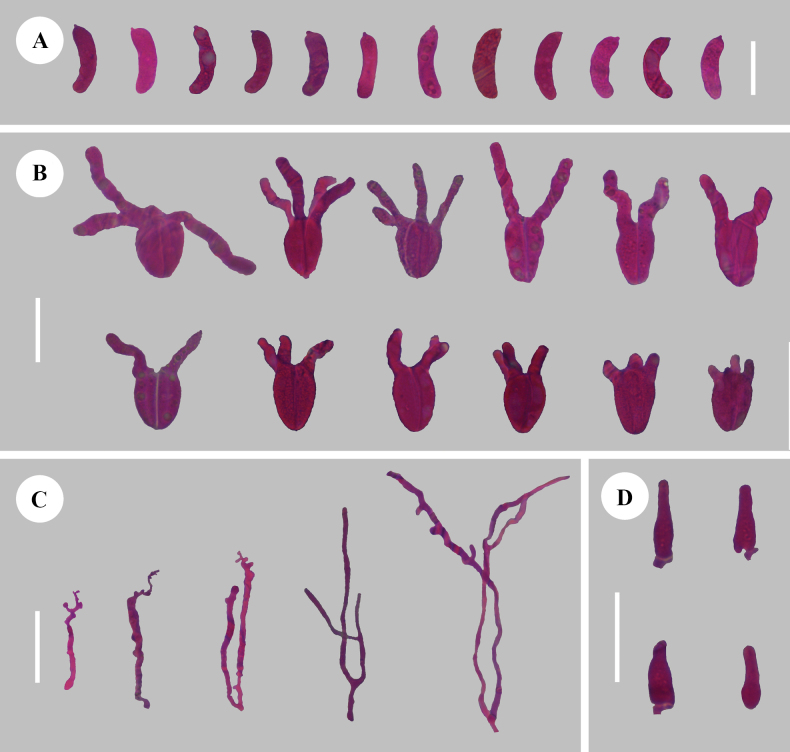
Sections of hymenium of *Alloexidiopsisxantha* (holotype, CLZhao 25093) **A** basidiospores **B** basidia **C** hyphidia **D** cystidia. Scale bars: 20 µm (**A–D**); 10 × 100 Oil.

## ﻿Discussion

In the present study, four new species *Ad.albomarginata*, *Ad.punctata*, *Al.grandinea* and *Al.xantha* are described, based on the phylogenetic analyses and morphological characteristics.

**Figure 15.**
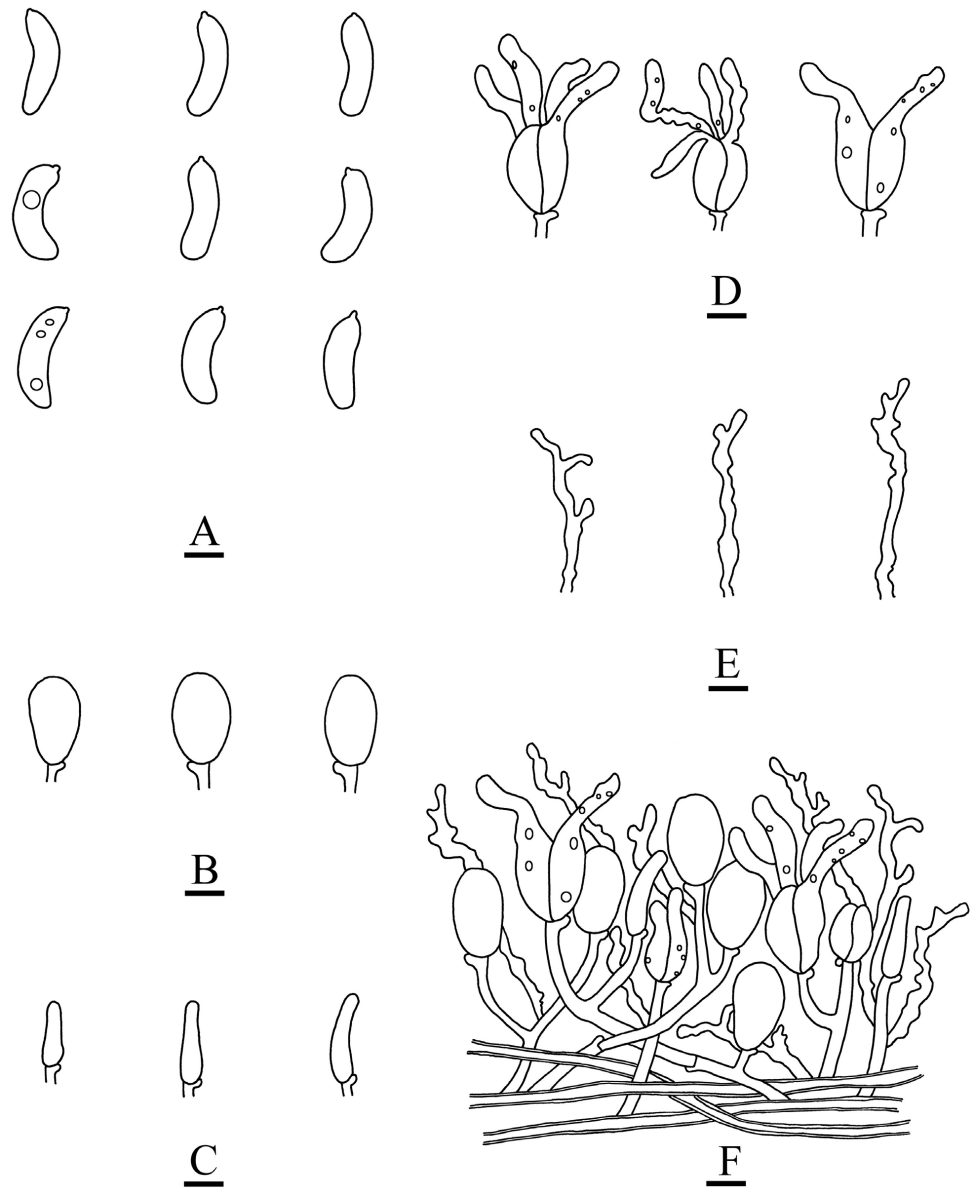
Microscopic structures of *Alloexidiopsisxantha* (holotype, CLZhao 25093) **A** basidiospores **B** basidioles **C** cystidia **D** basidia **E** hyphidia **F** part of the vertical section of hymenium. Scale bars: 10 µm (**A–F**).

The corticioid species of the order Auriculariales are traditionally placed in *Eichleriella*, *Exidiopsis* and *Heterochaete* according to the morphological characteristics (Liu et al. 2022). On the basis of the erection of six new genera as *Adustochaete*, *Alloexidiopsis*, *Amphistereum*, *Crystallodon*, *Proterochaete* and *Sclerotrema*, they were placed in the corticioid species and three previously known genera were reinstated, for example, *Hirneolina*, *Heteroradulum* and *Tremellochaete* (Malysheva and Spirin 2017; Alvarenga et al. 2019; Alvarenga and Gibertoni 2021; Liu et al. 2022). A multilocus-based phylogeny with a wider sampling of various morphological groups in Auriculariales is urgently needed to achieve a more natural classification of this order, as in other orders within Agaricomycetes (Wang et al. 2021).

Phylogenetically, based on the combined ITS+nLSU sequence data (Fig. 1), it demonstrated that the four new species were nested in the genera *Adustochaete* and *Alloexidiopsis* within the order Auriculariales. Based on ITS topology tree (Fig. 2), *Ad.albomarginata* was retrieved as a sister to *Ad.yunnanensis* and the species *Ad.punctata* was sister to *Ad.rava*. However, *Ad.yunnanensis* differs from *Ad.albomarginata* by its grandinioid hymenial surface, longer basidia (25–47.5 × 8.5–14 µm) and smaller cystidia (17.5–24.5 × 3.5–5.8 µm; Li and Zhao (2022)). *Ad.rava* can be distinguished from *Ad.punctata* by its spined, sharp-tipped hymenial surface, smaller basidia (14.9–16.2 × 9.7–10.1 μm) and basidiospores (10.2–13.6 × 4.6–5.9 µm; Hyde et al. (2020)). Based on ITS topology tree (Fig. 3), *Al.grandinea* was retrieved as a sister to *Al.schistacea* and *Al.xantha* formed a monophyletic lineage. However, *Al.schistacea* differs from *Al.grandinea* by its smooth hymenial surface and longer basidia (15–20 × 7–10 µm; Liu et al. (2022)).

Morphologically, two new species *Adustochaetealbomarginata* and *Ad.punctata* resemble four similar species in the genus *Adustochaete*, *Ad.interrupta* Spirin & Malysheva, *Ad.nivea*, *Ad.rava* and *Ad.yunnanensis*. A morphological comparison between two new *Adustochaete* species and four similar species are presented in Table 2. Two new species *Al.grandinea* and *Al.xantha* are similar to five species in the genus *Alloexidiopsis*, *Al.australiensis* S.L. Liu, Z.Q. Shen & L.W. Zhou, *Al.calcea* (Pers.) L.W. Zhou & S.L. Liu, *Al.nivea* (J.J. Li & C.L. Zhao) L.W. Zhou & S.L. Liu, *Al.schistacea* and *Al.yunnanensis*. A morphological comparison between two new *Alloexidiopsis* species and six similar species are presented in Table 3.

**Table 2.** A morphological comparison between two new *Adustochaete* species and four similar species in the genus *Adustochaete*.

Species name	Hymenial surface	Hyphae	Cystidia	Basidia	Basidiospores	References
***Adustochaetealbomarginata***	Smooth/ Cream to buff	Thin-walled, unbranched	Subclavate to fusiform; 23.5–48.5 × 10–13.5 µm	Ellipsoid to ovoid, two to four-celled; 17–24.5 × 11–16.5 µm	Subcylindrical to allantoid; 12–17.5 × 6.5–9 µm	**Present study**
*Adustochaeteinterrupta*	Smooth/ Light ochraceous-grey to brownish	Thin-walled	Clavate to fusiform; 45–96 × 6–13.5 µm	Narrowly ovoid to obconical, four-celled; 15.1–24 × 9.1–11.8 µm	Broadly cylindrical; 11.3–14.3 × 5.7–6.2 µm	Alvarenga et al. (2019)
*Adustochaetenivea*	Sharp-tipped spines/ White	Thin-walled	—	Narrowly ovoid to obconical, four-celled; 14.9–16.2 × 9.7–10.1 μm	Cylindrical; 10.2–13.6 × 4.6–5.9 µm	Hyde et al. (2020)
***Adustochaetepunctata***	Smooth/ Punctate, white to cream	Thin-walled, unbranched	Subcylindrical to clavate; 15.5–23.5 × 5.5–7.5 µm	Ellipsoid to ovoid, two to four-celled; 17–25 × 16.5–21 µm	Subcylindrical to allantoid; 13.5–18 × 6–8.2 µm	**Present study**
*Adustochaeterava*	Sharp-tipped spines/ Pale to dark grey	Thin-walled	Clavate to fusiform; 27–52 × 4–8 µm	Narrowly ovoid to obconical, four-celled; 10.8–15.2 × 7.3–10 µm	Cylindrical; 10.2–13.7 × 3.8–4.7 µm	Alvarenga et al. (2019)
*Adustochaeteyunnanensis*	Grandinioid/ Dark greyish to brownish	Thin-walled, branched	Clavate to fusiform; 17.5–24.5 × 3.5–5.8 µm	Narrowly ovoid to obconical, four-celled; 25–47.5 × 8.5–14 µm	Narrow cylindrical to allantoid; 12–20 × 5–7 µm	Li and Zhao (2022)

**Table 3.** A morphological comparison between two new *Alloexidiopsis* species and six similar species in the genus *Alloexidiopsis*.

Species name	Hymenial surface	Hyphae	Cystidia	Basidia	Basidiospores	References
*Alloexidiopsisaustraliensis*	Smooth, covered by sterile spines/ Cream to pale orange	Thin-walled, branched	Cylindrical, ventricose; 21.5–24.5 × 9.5–12 µm	Ellipsoid to ovoid, four-celled; 18–21 × 13–18 µm	Cylindrical to broadly cylindrical; 13–25 × 7–11 µm	Li et al. (2022b)
*Alloexidiopsiscalcea*	Granulose to pruinose/ Greyish-white to light ochraceous	Thin-walled, branched	—	Obovate to clavate, two to four-celled; 14–25 × 9.5–15 µm	Allantoid to cylindrical, sometimes helicoid; 12–18 × 5–7 µm	Wells (1961)
***Alloexidiopsisgrandinea***	Grandinioid/ Buff to slightly yellowish	Thin-walled, branched	Fusiform; 20–42.5 × 5.5–9.5 µm	Ellipsoid to ovoid, two to four-celled; 12.5–14.5 × 9–11.5 µm	Cylindrical to allantoid; 10–12.3 × 5–5.8 µm	**Present study**
*Alloexidiopsisnivea*	Smooth/ White to slightly cream	Thin-walled, unbranched	Tubular; 15–34 × 2.5–7 µm	Narrowly ovoid to obconical, two to four-celled; 9–19 × 8–15 µm	Allantoid; 6.5–13.5 × 2.7–5.5 µm	Li et al. (2022a)
*Alloexidiopsisschistacea*	Smooth/ Greyish	Thin-walled, branched	Cylindrical; 25–50 × 4–6 µm	Ellipsoid to ovoid, four-celled; 15–20 × 7–10 µm	Cylindrical to broadly cylindrical; 9.5–11 × 4.5–5.5 µm	Liu et al. (2022)
*Alloexidiopsissinensis*	Grandinoid/ Yellowish-brown to rose to slightly purple	Thin- to thick-walled, branched	Cylindrical; 11.5–15.5 × 3–5.5 µm	Ellipsoid to ovoid, two to four-celled; 16–22 × 7.5–10 µm	Allantoid; 14.5–23 × 4.5–6.5 µm	Dong et al. (2024)
***Alloexidiopsisxantha***	Smooth/ Cream to slightly buff	Thin- to thick walled, branched	Subcylindrical to subconiform; 12.5–17.5 × 3.5–6 µm	Ellipsoid to ovoid, obconical; 18–20.5 × 12–15.5 µm	Allantoid, sickle-shaped; 20–24 × 5–6.2 µm	**Present study**
*Alloexidiopsisyunnanensis*	Odontoid/ White to smoke grey	Thin-walled, unbranched	Clavate to fusiform; 13–35 × 2–6 µm	Narrowly ovoid to obconical, two to three-celled; 28–41 × 9–14 µm	Cylindrical; 17–24 × 5–8 µm	Guan et al. (2020)

In the ecological distribution, both genera species are not an extensively studied group, distributed worldwide and mainly found on hardwood (Alvarenga et al. 2019; Liu et al. 2022). The species of *Adustochaeteinterrupta* Spirin & Malysheva was found in Mexico, *Ad.nivea* was described in Brazil, *Ad.rava* was found in Brazil and *Ad.yunnanensis* was found in China. The species of *Alloexidiopsisaustraliensis* was found in Australia, *Al.calcea* was found in Germany and *Al.nivea*, *Al.schistacea*, *Al.sinensis* and *Al.yunnanensis* were found in China.

Fungi are one of the most diverse groups of organisms on Earth and play a crucial role in ecosystem processes and functions (Hyde 2022). New DNA sequencing techniques have revolutionised the studies of fungal taxonomy and diversity, in which about 150 k species of fungi have been described (Hyde 2022). In recent years, the wood-inhabiting fungi are an extensively studied group of Basidiomycota, which includes a number of poroid, smooth, grandinoid, odontioid and hydnoid basidiomata in China (Wu et al. 2022a, 2022b; Dong et al. 2023a, 2023b; Guan et al. 2023; Liu et al. 2023; Mao et al. 2023; Yang et al. 2023, 2024; Deng et al. 2024; Li et al. 2024; Luo et al. 2024; Zhang et al. 2024; Zhao et al. 2024; Zhou et al. 2024). In the past several years, many corticioid species have been reported and described in the order Auriculariales (Malysheva and Spirin 2017; Alvarenga et al. 2019; Spirin et al. 2019a, 2019a; Alvarenga and Gibertoni 2021; Li et al. 2022a, 2022b; Li and Zhao 2022; Liu et al. 2022), but many new taxa have not yet been discovered. Thus, the corticioid species diversity of the order Auriculariales is still not well known in China, especially in the subtropical and tropical areas. In the present study, four new species, *Ad.albomarginata*, *Ad.punctata*, *Al.grandinea* and *Al.xantha* were found and reported. This paper enriches our knowledge of fungal diversity in the order Auriculariales. We anticipate that more undescribed corticioid taxa will be discovered throughout China after extensive collection combined with morphological and molecular analyses.

### ﻿Key to the known species of *Adustochaete* worldwide

**Table:** 

1	Hymenial surface smooth	**2**
–	Hymenial surface grandinioid	**4**
2	Basidia > 16.5 µm wide	***Adustochaetepunctata***
–	Basidia < 16.5 µm wide	**3**
3	Basidiospores > 6.5 µm wide	***Adustochaetealbomarginata***
–	Basidiospores < 6.5 µm wide	***Adustochaeteinterrupta***
4	Cystidia absent	***Adustochaetenivea***
–	Cystidia present	**5**
5	Basidiospores > 5 µm wide, basidia > 16 µm long	***Adustochaeteyunnanensis***
–	Basidiospores < 5 µm wide, basidia < 16 µm long	***Adustochaeterava***

### ﻿Key to the known species of *Alloexidiopsis* worldwide

**Table:** 

1	Basidiospores allantoid	**2**
–	Basidiospores cylindrical	**6**
2	Hymenial surface smooth	**3**
–	Hymenial surface grandinoid, granulose to pruinose	**4**
3	Basidiospores > 13.5 µm long, cystidia subcylindrical to subconiform	***Alloexidiopsisxantha***
–	Basidiospores < 13.5 µm long, cystidia tubular	***Alloexidiopsisnivea***
4	Cystidia absent	***Alloexidiopsiscalcea***
–	Cystidia present	**5**
5	Cystidia > 5.5 µm wide	***Alloexidiopsisgrandinea***
–	Cystidia < 5.5 µm wide	***Alloexidiopsissinensis***
6	Basidia > 28 µm long, cystidia clavate to fusiform	***Alloexidiopsisyunnanensis***
–	Basidia < 28 µm long, cystidia cylindrical	**7**
7	Basidiospores > 11 µm long, cystidia < 25 µm long	***Alloexidiopsisaustraliensis***
–	Basidiospores < 11 µm long, cystidia > 25 µm long	***Alloexidiopsisschistacea***

## Supplementary Material

XML Treatment for Adustochaete albomarginata

XML Treatment for Adustochaete punctata

XML Treatment for Alloexidiopsis grandinea

XML Treatment for Alloexidiopsis xantha

## ﻿Additional information

### Author ORCIDs

Junhong Dong https://orcid.org/0000-0001-8740-0805

Yonggao Zhu https://orcid.org/0009-0008-5341-3796

Chengbin Qian https://orcid.org/0009-0003-5329-1016

Changlin Zhao https://orcid.org/0000-0002-8668-1075

### Data availability

All of the data that support the findings of this study are available in the main text.
